# Feasibility of using diamond-like carbon films in total joint replacements: a review

**DOI:** 10.1007/s10856-024-06814-x

**Published:** 2024-08-13

**Authors:** Anurag Roy, Annette Bennett, Lisa Pruitt

**Affiliations:** Department of Mechanical Engineering, University of California, Berkeley, CA USA

## Abstract

Diamond-like Carbon (DLC) has been used as a coating material of choice for a variety of technological applications owing to its favorable bio-tribo-thermo-mechanical characteristics. Here, the possibility of bringing DLC into orthopedic joint implants is examined. With ever increasing number of patients suffering from osteoarthritis as well as with the ingress of the osteoarthritic joints’ malaise into younger and more active demographics, there is a pressing need to augment the performance and integrity of conventional total joint replacements (TJRs). Contemporary joint replacement devices use metal-on-polymer articulations to restore function to worn, damaged or diseased cartilage. The wear of polymeric components has been addressed using crosslinking and antioxidants; however, in the context of the metallic components, complications pertaining to corrosion and metal ion release inside the body still persist. Through this review article, we explore the use of DLC coatings on metallic bearing surfaces and elucidate why this technology might be a viable solution for ongoing electrochemical challenges in orthopedics. The different characteristics of DLC coatings and their feasibility in TJRs are examined through assessment of tribo-material characterization methods. A holistic characterization of the coating-substrate interface and the wear performance of such systems are discussed. As with all biomaterials used in TJRs, we need mindful consideration of potential in-vivo challenges. We present a few caveats for DLC coatings including delamination, hydrophobicity, and other conflicting as well as outdating findings in the literature. We recommend prudently exploring DLC films as potential coatings on metallic TJR components to solve the problems pertaining to wear, metal ion release, and corrosion. Ultimately, we advise bringing DLC into clinical use only after addressing all challenges and concerns outlined in this article.

**Graphical Abstract:**

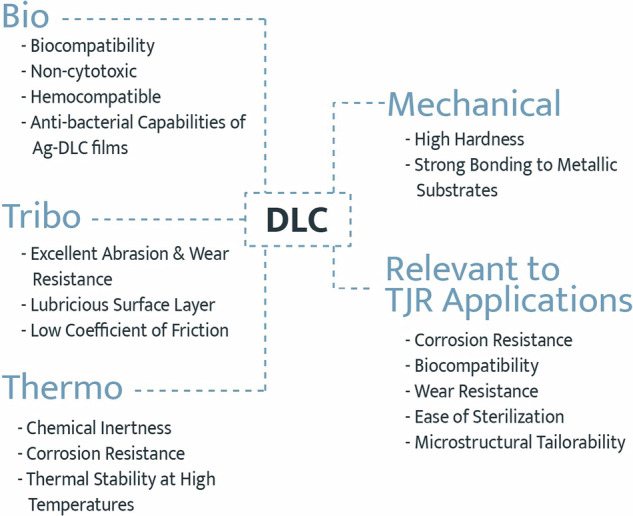

## Societal challenge: osteoarthritis

Osteoarthritis is increasingly afflicting more and more people with as many as 32.5 million people suffering from it in the US alone [1]. While initial remedies may include engaging in more physical activities and exercise, attempts to lose weight, medications, or even regular injections, a severely degraded osteoarthritic joint ultimately needs a total joint replacement (TJR) [2]. This is especially important given the preponderance of osteoarthritic knees in the general population, afflicting as many as 10% of men and 13% of women above the age of 60 years [3]. An osteoarthritic hip afflicts about 20% of people above the age of 65 years [4]. Further, the market demand for TJRs (which is the ultimate solution to extreme cases of osteoarthritic joints) is predicted to grow at staggering rates in the upcoming yeas [5]. While TJRs substitute the original joint function, they too are an assembly of articulating mechanical components which are prone to failure and thus, have a finite lifetime. Consequently, the revision burden for total knee replacements (TKRs) stands at ~10% at the 15-year mark while that of total hip replacements (THRs) is at ~7% at the decade mark [6, 7]. An additional complication is the duration of full recovery spanning over a year after knee revision surgeries and between 12–18 months for hip revisions [8].

## Total joint replacements: types, components, and materials

**Fig. 1:**
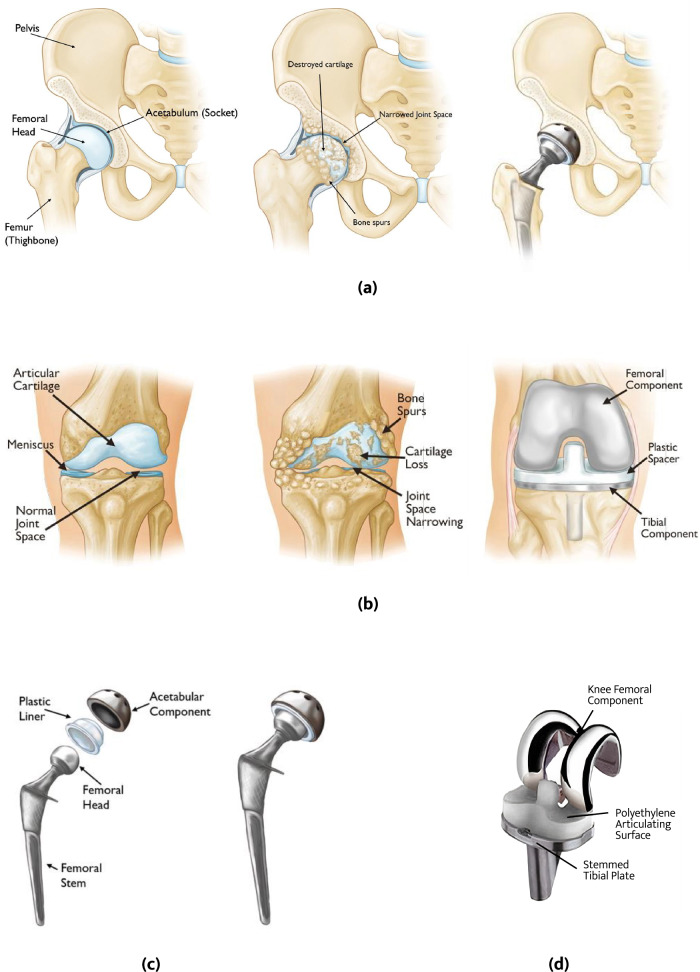
Healthy and unhealthy hip and knee joints, THR and TKR shown in (**a**) and (**b**) respectively, and the individualcomponents constituting THR and TKR illustrated in (**c**) and (**d**) respectively. Adapted from [6, 102, 103]

The TJRs of interest in this review encompass the total hip replacement (THR) and total knee replacement (TKR), which act as artificial substitutes for the natural hip and knee joints respectively. Figure 1a shows images of a healthy hip, an osteoarthritic hip, and a contemporary total hip replacement alongside the location where they are implanted. Similarly, Fig. 1b demonstrates the same set of images for the healthy knee, osteoarthritic knee, and total knee replacement. In Fig. 1c, d, the individual components constituting the total hip and total knee replacement are illustrated. While different material combinations [9] have been used for these individual components, the predominant materials of interest are as follows:

### THR

Femoral head comprising CoCr (CoCrMo) alloy, zirconia, alumina, or Oxinium. Plastic liner made from Ultra-high Molecular Weight Polyethylene (UHMWPE) and acetabular shell composed of a Ti alloy (Ti_6_Al_4_V). Femoral stems utilize Ti_6_Al_4_V or CoCrMo alloys.

### TKR

Femoral component prepared from CoCr alloy, the plastic spacer comprising UHMWPE, and the underlying tibial component utilizing a Ti alloy as aforementioned.

There exist varied kinds of joint replacement systems including metal-on-polymer (MoP), metal-on-metal (MoM), ceramic-on-ceramic (CoC), and ceramic-on-polymer (CoP). The primary focus in this review will be on MoP given their predominant use in orthopedics (51%) followed by MoM (35%) and the rest being CoC and CoP [9–11]. The MoP gold standard remains CoCr articulating against UHMWPE.

## Challenges plaguing present-day TJRs

One of the main challenges to the current designs and materials arises from the fact that conventional designs and materials for TJRs were originally meant for an older demographic with a majorly sedentary lifestyle. Yet, the trend in orthopedics across hip and knee replacement is toward younger demographics with more active lifestyles [12]. This puts additional loading demand on the TJR and affects performance requirements.

Another problem arises from the years of usage after the implantation of metallic components inside the aqueous environment of the body (making them prone to corrosion) and the variable loading on the joint space (inducing fatigue damage), continuous articulation during any locomotion (causing wear), and sudden impacts during physical activities (leading to possible fracture). Thus, the chances of mechanical failure through any of these modalities is greatly enhanced, i.e. through corrosion, fracture, fatigue, and wear [13, 14]. There are also secondary failure modalities combining these, such as fatigue-induced wear, fatigue-induced fracture, and stress-corrosion cracking.

In this review, the primary focus is confined to solving the challenges of corrosion and metal ion release associated with wear of the metallic component used in TJR. Metal debris and ion release leads to metallosis, inflammation, hypersensitivity, and the possibility of pseudo tumors inside the body [15–17].

A specific clinical challenge is related to metallic components in the femoral side of total hip replacements. Ti is generally used for the femoral stem owing to its excellent fatigue resistance as well as its propensity to mitigate stress shielding while affording mechanisms for osseointegration. Unfortunately, Ti alloys suffer from poor wear performance and fretting corrosion. For these reasons, the Ti alloy is not used as a bearing surface in a TJR space and instead the Ti stem is often coupled to a Co-Cr head through a Morse taper [14]. Such modular systems offer mechanical advantages but render the system prone to local galvanic or crevice corrosion processes [18, 19]. However, with a robust coating applied on its surface, Ti potentially becomes a candidate for the femoral head [20]. This would enable the use of a monolithic Ti-alloy femoral component that could mitigate wear and corrosion mechanisms.

## Potential solution: diamond-like carbon overcoats

One plausible solution to the aforementioned problems is the utilization of Diamond-like Carbon (DLC) [21, 22] coatings on the metallic component of the orthopedic bearing system. This would entail its application as an overlay on CoCr to potentially solve the corrosion and metal-ion release problems as well as potential to make Ti a candidate for articulating elements such as the femoral head. A holistic assessment of DLC technology for creating a protective overlay that mitigates wear, corrosion, and metal ion release is the overarching theme of this review article.

**Fig. 2:**
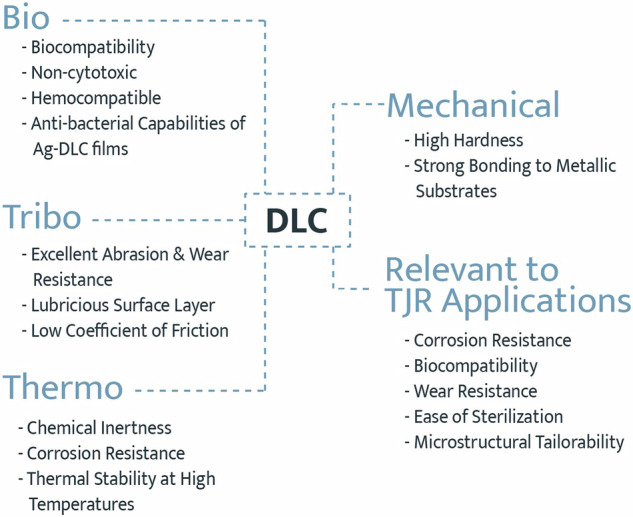
DLC’s bio-tribo-thermo-mechanical characteristics, which make it an ideal candidate for use in TJR applications. Adapted from [24]

The interest in DLC in TJR applications arises from its unique bio-tribo-thermo-mechanical performance in multiple application domains [23–40]. This combination of attributes includes biocompatibility; wear resistance, high hardness and low coefficient of friction; thermal stability and chemical inertness; strong adhesion to metallic substrates; as well as the ability for tailored, through-thickness microstructures [21, 26, 28, 32, 41–47]. Figure 2 summarizes the favorable features exhibited by DLC films for use in TJR.

## DLC’s applicability to TJR systems

For materials being considered for TJRs, it is paramount that they be biocompatible and can be implanted inside the body without causing any adverse effects in patients. DLC coatings are generally considered to be bioinert and biocompatible. Studies addressing cytotoxicity, cell-growth, mutagenicity, hemocompatibility, thrombogenicity, and cell-growth indicate that DLC coatings are viable candidates for implantable biomaterial applications [21, 22, 29, 32, 48–55].

DLC coatings show promise for the mitigation of corrosion processes in the body. Notably, DLC films on orthopedic grade CoCrMo in a simulated body fluid environment have been shown to corrode at a rate of 4–5 orders of magnitude lower than an uncoated counterpart [56]. Other tribo-corrosion studies using DLC on CoCrMo or Ti alloy or stainless steel substrates in biological environments have also affirmed their corrosion resistance [32, 39, 56–58].

Further, positive research results on DLC coatings for orthopedic and maxillofacial screws as well as coronary artery stents gives further credence to their potential as an overlay to preclude metal ion release and corrosion along with chronic inflammatory response spanning prolonged durations [22, 55, 59, 60]. The addition of Ag to form composite Ag-DLC films enhances the films’ anti-bacterial capabilities, which favor the possibility of it being used in TJRs [61, 62].

DLC-coated CoCr and Ti alloys have performed well tribologically against UHMWPE and evince a significant reduction in wear in TJRs [26, 39, 63–66]. DLC-coated metallic components could reduce metal ion release into the body and thereby prevent complications arising from the adverse biological reactions they elicit as there would be no contact between the polymer bearing and the metallic components of the TJRs. Thus, it is hypothesized that DLC would reduce the severity of the ongoing challenge with metal-ion release in orthopedic devices.

The thermal stability of DLC coatings assures that sterilization will not be cause for concern in orthopedic components. Nanoscale films of DLC maintain their thermo-mechanical properties and structural integrity up to temperatures of 250 ˚C [67]. Given that autoclaving (121 ˚C) and ethylene oxide (EtO) sterilization (~60 ˚C) transpire at much lower temperatures, it can be safely presumed that these treatments will not cause thermal deterioration of the DLC coatings. In that same vein, given the exceptional chemical inertness of DLC films [41], it is unlikely to be affected by the chemical agents used during EtO sterilization.

## Multilayered structure of DLC

One particularly interesting facet of DLC films is their microstructural tailorability [43, 68, 69]. This is especially applicable to DLC films deposited using two specialized techniques, namely, filtered cathodic vacuum arc (FCVA) and plasma-based immersion methods (PBIIID). In both cases, the films exhibit a multi-layered structure. A typical DLC film produced through these two methods is comprised of three layers: a surface layer with a higher percentage of *sp2* hybridized carbon (more graphite-like) followed by a bulk layer which is extremely hard owing to a predominantly *sp*^*3*^ hybridized state (more diamond-like), and an intermixing layer [43, 68].

**Fig. 3:**
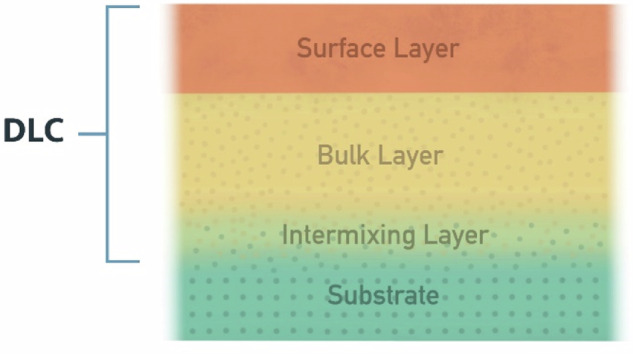
DLC’s multilayered structure

The soft and lubricious surface layer is responsible for the low coefficient of friction and smooth articulation against the counter-surface in tribologically challenging applications, such as bearing surfaces in TJRs. On the other hand, the core bulk layer provides thermal stability, chemical inertness, hardness, as well as wear and corrosion resistance. The intermixing layer acts as a transition layer between the bulk of the carbon film and the underlying substrate, aiding interfacial adhesion, and precluding delamination by reducing the adverse effect of sharp stress-strain gradients at the interface [24]. Instead, the intermixing layer provides a smooth gradient between the two radically different layers, i.e., carbon and metal. The multilayered structure of DLC films is best visualized in Fig. 3.

## DLC synthesis

There is a plethora of synthesis techniques to deposit DLC coatings atop metallic substrates, such as sputtering, ion beam-assisted deposition, plasma-enhanced chemical vapor deposition, plasma-based immersion methods, and filtered cathodic vacuum arc. Roy et al. provide a comprehensive review of the specialized coating techniques applicable to DLC film synthesis [24]. Two techniques stand out for the exceptionally hard and dense coatings they produce concomitant with precise microstructural control of the DLC film. These are the plasma-based immersion ion implantation and deposition (PBIIID) and filtered cathodic vacuum arc (FCVA). Sputtered films are commonly utilized owing to their quality, functionality, and cost efficiency.

**Fig. 4:**
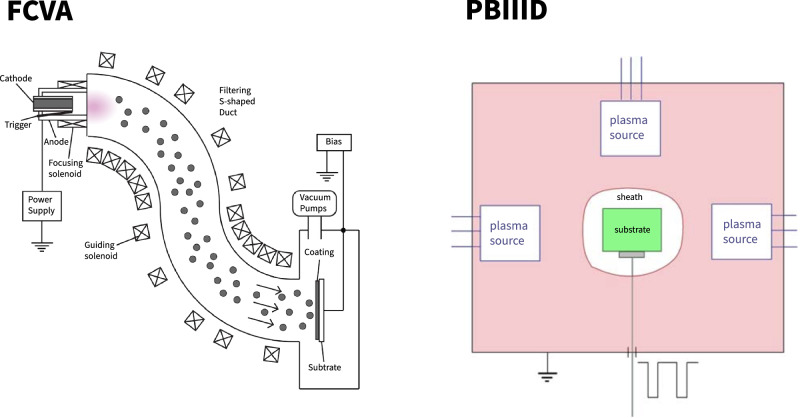
Coating of 2-D substrates supported by FCVA and 3-D components by PBIIID. Adapted from [104, 105]

FCVA and PBIIID are strikingly similar from the deposition physics standpoint. The difference between them is that the physical object is placed on the path of a plasma stream in FCVA (thereby coating a plane surface) rather than being immersed in a plasma cloud as is the case with PBIIID (3D object coating). The 3D coating ability offered by PBIIID makes it the technique of choice over FCVA, since most TJR components have 3D geometries. However, FCVA offers the ability to co-deposit carbon along with other metal ions to form DLC composite coatings with enhanced functionalities which is not possible in PBIIID. An illustration conveying the subtle differences in the setups for FCVA and PBIIID is presented in Fig. 4. We believe that the best deposition method would be a PBIIID chamber that sources the individual plasma streams from multiple sides using multiple FCVA-systems adjoining the PBIIID chamber, thereby integrating FCVA with PBIIID to maximize the synergistic benefits of both these specialized DLC coating methods. This integrated setup could be applied for coating TJR components with DLC as discussed in this review.

## Tribo-material characterization of DLC on TJRs

The tribo-material landscape of DLC overcoats on metallic components must be fully explored before making a final recommendation concerning TJRs. Material characterization should include visualization of the cross-section of the coating-substrate interface using Scanning Electron Microscopy (SEM) or High-Resolution Transmission Electron Microscopy (HRTEM). These techniques identify key morphological attributes of the films such as uniformity, conformity, continuity, and surface roughness [23, 44, 70].

Electron Energy-Loss Spectroscopy (EELS) [44, 68, 69, 71, 72] is typically utilized to characterize the intermixing layer which is intrinsic to FCVA- and PBIIID-deposited DLC films. EELS provides a chemical fingerprint of the elements present at specific locations on the cross-section and is beneficial in ascertaining the extent of carbon penetration into the underlying metallic substrate. The depth of carbon penetration into the underlying matrix defines the intermixing layer thickness and correlates with the adhesion strength as well as reduction in the possibility of delamination at the coating-substrate interface [24].

Tribological characterization is broadly classified into three categories: (a) wear and friction tests, which include both pin-on-disk (PoD) bench tests as well as joint simulator testing, (b) post-wear fractography, and (c) surface roughness measurements.

### Wear and friction tests

Historically, tribological wear evaluations were performed on simple PoD bench setups using a circular unidirectional motion test. However, this method fails to emulate the multidirectional cross-shear motion that is commonly experienced in TJRs [73]. Rudimentary PoDs therefore remain constrained to preliminary material assessments and exploring simple wear behavior of material systems.

Joint simulators were developed to emulate the complex kinematics and loading of the gait cycle. Such systems allow for assessment of various combination of designs, materials, tribological parameters, and lubrication schemes for TJRs [32, 74–82]. Complex joint simulators combine multiple motions and multidirectional loads, making them more pertinent for final-stage design and material selections but these expensive (and time consuming) tests fail to isolate the fundamental mechanisms of wear at the constituent level.

Alternatively, multi-directional tribometers capture a wide variety of motions and loadings, starting from simple pin-on-disk in planar sliding to complex rolling and rotation motions in ball-on-flat contacts under clinically relevant conditions [83–85]. The tribo-couple of relevance is UHMWPE articulating against DLC-coated metallic TJR components (CoCr or Ti alloy) in all tribological experiments.

Tribological tests enable characterization of coefficients of friction [32] as well as the adhesion behavior of the DLC coatings. Thicker intermixing layers inherent in certain deposition techniques such as FCVA and PBIIID lead to stronger adhesion at the coating-substrate interface [46, 86, 87]. Enhanced adhesion of the coating reduces the likelihood of delamination and premature failure in TJRs. Moreover, precluding delamination mitigates metal ion release from the underlying substrate and its consequent toxic effects in the body.

### Post-wear fractography

**Fig. 5:**
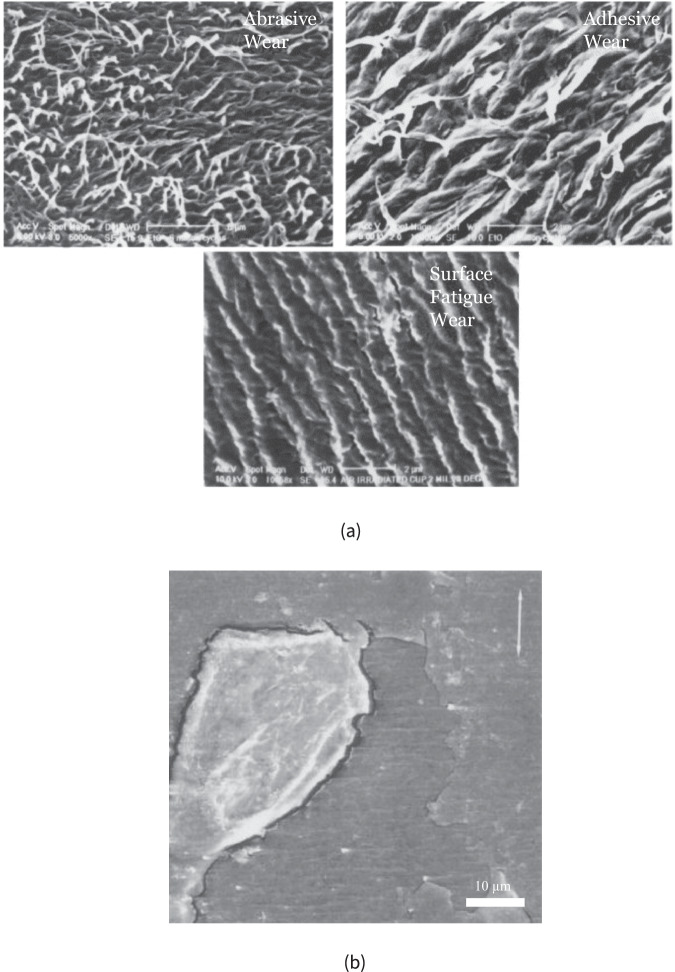
Post-wear fractographic analysis using SEM demonstrating (**a**) abrasive, adhesive, and surface fatigue wear [73], as well as (**b**) delamination wear [106]. Adapted from [73, 106]

Fractography provides insight into coating failure modality and mechanisms of wear. Post-articulation SEM of worn components has revealed a number of wear mechanisms including abrasive, adhesive, surface fatigue, and delamination [61, 73, 86] as illustrated in Fig. 5. SEM fractography of the DLC-coated metallic components is requisite in assessing whether delamination or surface damage is of concern in TJRs. Delamination or spalling of the coating is detrimental as particulate debris can lead to metallosis and metal ion release in the body.

### Surface roughness measurements

The final step in completing the tribo-material characterization of TJRs involves measuring the surface roughness of the DLC-coated TJR components via Atomic Force Microscopy (AFM) or Surface Force Microcopy (SFM) [44, 70, 88–91].

**Fig. 6:**
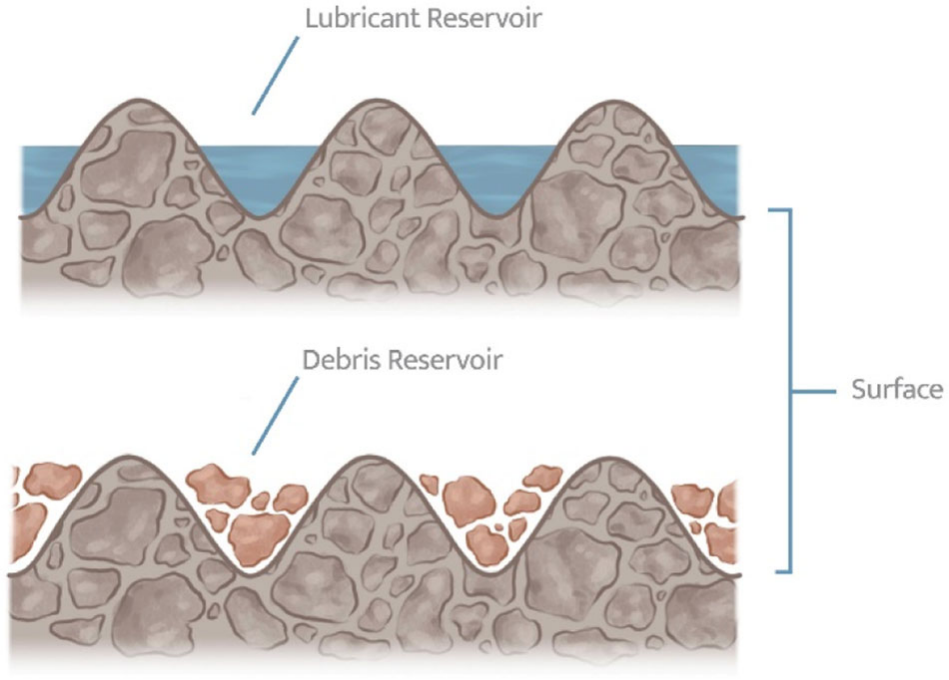
Surface roughness as a lubricant reservoir or debris reservoir hypothesis

The effect of surface roughness on the tribological performance of DLC films in TJRs has two possible outcomes which are posited here. First, that the higher surface roughness of the sputtered DLC (sputtered DLC is much more prone to island-like growth in comparison to FCVA or PBIIID [44, 70, 88–91]) act as reservoirs for the lubricant during the articulation. During stages of limited lubricant supply to the joint space, the inherent reservoirs may assist in boundary lubrication, thereby reducing the coefficient of friction and the rate of wear occurring at the site of articulation. The second theory runs contrary to the previous one, claiming that the increase in surface roughness acts as a reservoir for storing wear debris, which would then go on to detrimentally affect the tribological performance by catalyzing three-body wear. A visual of these effects is illustrated in Fig. 6.

In order to assess requisite tribological performance of DLC coatings in orthopedics, the influence of surface roughness and lubrication regimes in the articulating joint space needs to be examined in future studies. Whether surface roughness acts as a reservoir of lubricant or third body wear debris will have serious implications on increasing/decreasing the resilience of DLC-coated metallic components in TJRs. Similarly, the correlation between adhesion and thickness of intermixing layer warrants further investigation. These findings will foretell the likelihood of clinical success for incorporating DLC technology in TJRs.

## Challenges with using DLC in TJRs

**Fig. 7:**
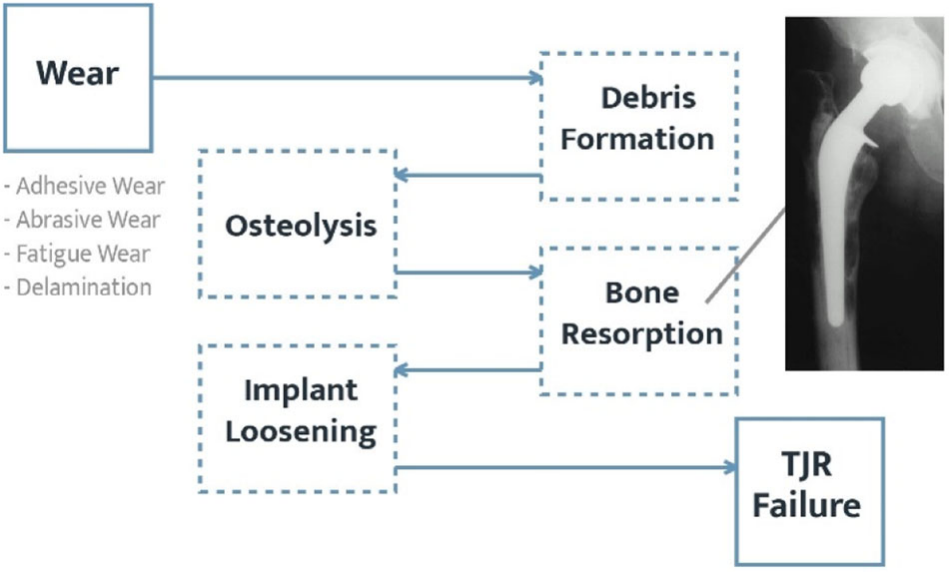
Various modalities of wear leading to the eventual failure of TJRs. Adapted from [107]

In the realm of orthopedics, contemporary formulations of ultra-high molecular weight polyethylene (UHMWPE) with optimized crosslinking and anti-oxidant technology have majorly rectified the wear-mediated osteolysis problem [92]. Yet, a further reduction in wear could enhance the lifetime of the TJRs by precluding implant loosening and associated failures. Figure 7 illustrates how wear debris from any source in a joint replacement can culminate in the eventual failure of the implant. Tribological testing which closely simulates the joint space conditions will need to be performed in order to assess DLC-coated metallic components articulating against modern UHMWPE components. This is important as previous wear studies reporting favorable wear outcomes between DLC and UHMWPE were conducted using older formulations of UHMWPE which are no longer clinically relevant [26, 39, 63–66].

A caution for DLC technology in orthopedics is that some of the tribological literature is not only outdated but also contradictory. Some earlier studies discourage the use of DLC in TJR systems, albeit on older formulations of UHMWPE. In some cases, there were increases in the wear rates for the DLC-coated CoCrMo-UHMWPE coupling [56, 93, 94]. Moreover, DLC coatings on Ti substrates showed no tribological improvements when articulating against UHMWPE in comparison to the gold standard of CoCr against UHMWPE under a multidirectional pin-on-disk test setup [95].

Some researchers have expressed concerns from their experiments on UHMWPE against DLC-coated ceramic substrates hypothesizing that the UHMWPE-DLC pair might exhibit high adhesion forces which is unfavorable from a tribological standpoint [96]. While the substrates were ceramic in nature, the high adhesion reported between UHMWPE and DLC is irrespective of the underlying substrate material and is cause for concern. Another study found no improvement in the corrosion resistance behavior exhibited by DLC-coated Ti in comparison to bare Ti substrates [97]. Finally, there have also been reports of low survivorship (54%) and aseptic loosening of DLC-coated Ti alloy components amongst THR patients 90 months after implantation [98].

While DLC shows promise and should be considered for further exploration in TJRs, it does come with some additional caveats. Designers need to be mindful of Hertzian contact stresses during tribological articulation [99]; peak stresses that coincide with the DLC coating-metal substrate interface could facilitate delamination, corrosion, and metal-ion release. To surmount this, the coating thickness must be optimized to ensure that the Hertzian stress peak does not coincide with the interface where propensity for delamination is greater.

Another concern is that DLC films are hydrophobic by nature and may interfere with natural lubrication schemes in-vivo [100, 101]. A more hydrophilic material is preferred in TJRs since it draws in the synovial fluid and facilitates lubrication in the joint space. A hydrophobic material’s incorporation could potentially exacerbate this tribological challenge and starve the joint space of much-needed natural lubricant. More work is needed in these areas to ascertain whether DLC technology is beneficial in the realm of modern orthopedic devices and material formulations.

## Conclusions and outlook

In summary, the authors of this review article recommend exploring the possibility of incorporating DLC films as potential coatings on metallic TJR components to solve the problems pertaining to wear, metal ion release, and corrosion. With the ever-increasing demand for arthroplasty in both the elderly and younger, more active patient populations, there is a need to find materials that can optimize long-term clinical performance of TJRs. DLC technology with its bio-tribo-thermo-mechanical attributes and tailorability potentially aids in this endeavor.
